# Development and Investigation of AlSi12-Based Composites Reinforced with Saffil^TM^ Fibers and Nickel-Coated Graphite Flakes

**DOI:** 10.3390/ma18225083

**Published:** 2025-11-08

**Authors:** Wojciech Wyrwa, Jakub Grzęda

**Affiliations:** Department of Lightweight Elements Engineering, Foundry and Automation, Faculty of Mechanical Engineering, Wrocław University of Science and Technology, 27 Wybrzeże Stanisława Wyspiańskiego St., 50-370 Wrocław, Poland; jakub.grzeda@pwr.edu.pl

**Keywords:** aluminum metal matrix composites, Saffil fibers, graphite flakes, mechanical properties, electrochemical impedance spectroscopy, corrosion resistance

## Abstract

Composites based on the AlSi12 aluminum alloy reinforced with Saffil^TM^ fibers (Composite I) and with both Saffil^TM^ fibers and nickel-coated graphite flakes (Composite II) were developed using the squeeze casting method in the fabrication process. The objective of this work was to evaluate the influence of the employed reinforcements on the mechanical properties and corrosion behavior of the obtained materials. To achieve this, investigations were conducted, including SEM analysis, flexural strength testing, Brinell hardness testing, linear sweep voltammetry (LSV) and electrochemical impedance spectroscopy (EIS). Corrosion measurements were performed in a 3.5% NaCl solution at room temperature. Mechanical investigations revealed a significant increase in flexural strength and hardness for Composite I compared to the plain matrix alloy. In contrast, Composite II’s flexural strength was reduced by the weakening effect of graphite flakes. Performance under bending improved by 46% for Composite I and 25% for Composite II compared to the AlSi12. The corrosion resistance of the tested materials followed the order AlSi12 > Composite I > Composite II. The LSV and EIS results indicate that the explanation for this may be differences in the properties of the protective oxide/hydroxide layer. Furthermore, SEM images showed a weak bond between nickel and graphite.

## 1. Introduction

AlSi12 is a widely used aluminum alloy, particularly in the automotive and aerospace industries. This is due to its favorable properties, such as a high strength-to-weight ratio, low density, excellent ductility, good thermal conductivity, low thermal expansion, and great castability. These characteristics make it ideal for lightweight structures. Some common applications include engine pistons, heat exchangers (e.g., car radiators, air conditioners), and compressor cylinder heads for air brake systems [1,2,3,4]. Additionally, AlSi12 has good corrosion resistance and the ability to naturally form a protective passivation layer [5]. This makes it suitable for use in marine and coastal environments as a structural material [6].

In order to improve mechanical properties and make aluminum alloys more suitable for specific technical applications and solutions, composites are developed. These materials include not only the main metal phase but also at least one reinforcing phase. This second phase usually has higher strength, stiffness or wear resistance. This group of engineering materials is known as aluminum metal matrix composites [4].

In recent years, many studies have been conducted using various materials as reinforcements. Unfortunately, in most of the works cited below there is no information about the influence of the used reinforcement on the change in corrosion properties between the utilized alloy and the obtained composites.

Common reinforcement materials used in composite matrices include ceramic particles such as silicon carbide (SiC), boron carbide (B4C), titanium carbide (TiC), cubic boron nitride (CBN), and aluminum oxide (Al_2_O_3_). The last material can also be introduced in the form of short fibers to enhance load-bearing capacity. These reinforcements are known to improve mechanical properties such as tensile strength, wear resistance, and hardness [7,8,9,10,11]. However, their effectiveness strongly depends on the type, morphology, and volume fraction of the reinforcing phase [12,13]. Additionally, several investigations have shown that the addition of graphite, particularly in flake form, positively influences wear resistance due to its solid lubricating properties [14,15]. Carbon fibers have also been employed to improve the strength and toughness of composites [16,17]. In recent years, more sustainable reinforcement strategies have emerged, involving the use of industrial by-products such as fly ash and red mud, which not only improve mechanical properties but also reduce production costs and environmental impact [18,19]. The improvement in the mechanical properties of the final material is attributed to several mechanisms, including load transfer, strong interfacial bonding, uniform distribution, which inhibits localized stress concentrations and the increase in overall hardness resulting from the incorporation of hard ceramic material into the aluminum matrix [20,21,22,23,24].

It should also be noted that, besides the type and form of the employed reinforcement, the production process affects the properties of the composite. Manufacturing methods are divided into two main groups: solid-state and liquid-state processes. The first category includes powder metallurgy and friction stir processing. In the case of liquid-state methods, techniques such as stir casting, squeeze casting, compocasting, and rheocasting are commonly utilized [4,25].

Sajjadi et al. [26] have investigated composites containing micro- or nano-sized Al_2_O_3_ particles, varying in the amount of reinforcement. The matrix material employed in this study was an A356 aluminum alloy, which contained 6 wt.% silicon. The authors fabricated composites with 1, 3, 5, and 10 wt.% Al_2_O_3_ macroparticles (20 µm) in the first group and with 1, 3, and 5 wt.% Al_2_O_3_ nanoparticles (50 nm) in the second group. The stir casting process, modified in a novel way and involving a three-step mixing procedure, was employed. The mechanical properties of the samples were tested and compared to pure matrix alloy. In each group of composites, an increase in the Brinell hardness value was observed. The highest values of this parameter were determined for materials containing 10 wt.% Al_2_O_3_ macroparticles (increasing by ca. 45% in relation to alloy) and 5 wt.% Al_2_O_3_ nanoparticles (increasing by ca. 44%). The addition of Al_2_O_3_ particles also improved compressive strength which demonstrated a different trend, with a more significant increase observed for the composites reinforced with nanoparticles. This means that the second group of investigated materials exhibited higher compressive strength values compared to composites with macroparticles. For instance, the highest compressive strength value was determined for the composite reinforced with 5 wt.% Al_2_O_3_ nanoparticles (increasing by ca. 161%). In the case of material strengthened with 10 wt.% Al_2_O_3_ macroparticles, the improvement was significantly lower, reaching ca. 93%. It is worth mentioning that aluminum matrix composites strengthened with Al_2_O_3_ are frequently used in advanced applications across industries such as automotive, military, aerospace, and electronics [26,27,28,29,30].

Venkat et al. [31] have developed a “hybrid metal matrix composite” strengthened by two types of ceramic particles. The researchers manufactured Al5052 (aluminum alloy) composites with Al_2_O_3_ and Si3N4 (silicon nitride), incorporating various weight fractions of Al_2_O_3_ (2, 4, and 6%) and a constant Si3N4 content of 3%. Stir casting methods were employed in the preparation process. The presented results demonstrate that the addition of reinforcement particles to the Al5052 matrix improved the mechanical properties compared to the base metal. The tensile strength was higher for all three composites, with the highest value observed for the material reinforced with 2% Al_2_O_3_ and 3% Si_3_N_4_, which had the lowest alumina content. In the other cases, the determined values were significantly lower. A similar trend was seen in the impact strength tests, where the energy values were higher for composites with 2% and 4% additional reinforcement.

In many studies [32,33,34,35,36,37,38], Naplocha et al. have investigated aluminum metal matrix composites, including ones reinforced with ceramic δ-Al_2_O_3_ Saffil fibers (10 and 20 vol.% of strengthening). The results of the conducted tests indicated an improvement in mechanical properties such as hardness and tensile strength at various temperatures. In the case of AlSi12 reinforced with 20 vol.% of fibers at the ambient temperature, an increase in tensile strength was observed, going from 150 MPa to 250 MPa compared to the pure matrix alloy. At a temperature of 300 °C the determined value was approximately 130 MPa, which is nearly double that of the unreinforced alloy. However, in the case of 10 vol.% of reinforcement, the measured values of this parameter were insignificantly higher compared to unstrengthened AlSi12. Furthermore, the hybrid composites reinforced with Al_2_O_3_ Saffil fibers and graphite in the form of flakes or fibers were obtained. The addition of carbon-based reinforcement improved wear resistance, but in the case of the flakes, the increase in the hardness value was less significant compared to the composite strengthened with fibers. To produce the abovementioned composites, the authors utilized the squeeze casting method.

Using powder metallurgy, El-Sayed Seleman et al. [39] have fabricated AA6016 aluminum alloy/graphite composites containing 5, 10, 15, and 20 wt.% graphite particles. The chemical composition of the matrix alloy included alloying elements such as Si (1.50 wt.%) and Mg (0.50 wt.%). To evaluate the microstructure and mechanical properties of the materials, the authors conducted SEM analysis, hardness tests, tensile strength tests, and pin-on-disk wear tests. SEM observations showed that even at a high reinforcement content, the graphite was distributed homogeneously within the alloy matrix. In the case of the composite containing 20 wt.% graphite, a Al_4_C_3_ (aluminum carbide) phase was detected. All graphite composites demonstrated higher hardness values compared to the base Al alloy, with the highest value observed for the 10 wt.% graphite composite. The wear resistance of the composites significantly improved as the reinforcement content increased, with the 20 wt.% graphite composite showing over ten times greater wear resistance than the matrix alloy. The tensile test results indicated that both the ultimate tensile strength and yield strength decreased as the graphite content increased.

As mentioned above, in the case of aluminum metal matrix composites reinforced with carbon materials or graphite flakes, a Al_4_C_3_ phase can form—especially at higher temperatures during manufacturing processes (>650 °C) [40]. Aluminum carbide worsens corrosion resistance of the composite material and can weaken its mechanical properties. Al_4_C_3_ may generate a galvanic couple with the aluminum matrix which hastens the corrosion process, particularly in electrolytic environments such as NaCl solutions [41]. Moreover, hydrolysis of this compound can further degrade the composite, decreasing its corrosion resistance through the production of. aluminum hydroxide and methane. Furthermore, Al_4_C_3_ can modify the microstructure of the composite, potentially resulting in micro-cracks and voids which can serve as initiation points for corrosion, thereby weakening the integrity of the material. Equations (1) and (2) below present the chemical reaction of the formation of Al_4_C_3_ and its hydrolysis, respectively [42].

(1) $$4\;\mathrm{Al}+3\;C\to {\mathrm{Al}}_{4}C_{3}$$

(2) $${\mathrm{Al}}_{4}C_{3}+12\;H_{2}O\to 4\mathrm{Al}{\left(\mathrm{OH}\right)}_{3}+3\;{\mathrm{CH}}_{4}↑$$

In order to avoid the formation of Al_4_C_3_ and to improve corrosion resistance, carbon-based reinforcements are coated with nickel or copper [14,15,40,43]. Additionally, this solution enhances the wettability which is crucial for effective bonding between the matrix and the reinforcement. In general, improvement in this particular property correlates with an enhancement in the quality of the bonding [44,45]. Employing the squeeze casting method, in [46], Bhavan et al. manufactured AA6061 composites reinforced with nickel-coated graphite (5 and 10 wt.%). The authors performed mechanical tests and the obtained results provided higher tensile strength (by ca. 96% and 101%) and Vickers hardness (by ca. 15% and 34%) compared to the unreinforced matrix. In both cases, the composite with the greater amount of flakes demonstrated the highest mechanical properties. However, in this work, there is no information about the corrosion behavior of the composites compared to the pure matrix alloy.

In this study, two types of AlSi12-based composite were manufactured—one reinforced with 20 vol.% Al_2_O_3_ Saffil fibers (Composite I) and the other reinforced with 20 vol.% Al_2_O_3_ Saffil fibers and 3 vol.% nickel-coated graphite flakes (Composite II). In the preparation process, the squeeze casting method was employed due to its advantages, such as improved bonding and reduced porosity, compared to other fabrication methods [4]. The mechanical properties of the tested samples were determined, including the Brinell hardness and flexural strength. Scanning electron microscopy (SEM) analysis was performed in order to present the structure of the obtained composites and to evaluate fractures after flexural strength testing. The mechanical investigations concerning aluminum metal matrix composites reinforced with saffil fibers [33,34,35,36,37,38,39] mainly focused on tensile strength, microstructural analysis and wear behavior. Therefore, in this work, flexural strength tests were selected to provide information that has been missing in the literature. Furthermore, the corrosion resistance in a 3.5% NaCl solution at room temperature was determined by employing linear sweep voltammetry (LSV) and electrochemical impedance spectroscopy (EIS). The results of the investigations were compared with the values determined for the matrix alloy. This study addresses a gap in the current state of the art by investigating the influence of used structural modifications on change in corrosion properties.

## 2. Materials and Methods

### 2.1. Materials

In this work, composites strengthened with Saffil fibers or nickel-coated graphite flakes were produced. Table 1 provides the most important information about the obtained materials and proposed notations employed in this study.

An AlSi12 (Prometal, Trzebinia, Poland) aluminum alloy was selected as a matrix material, and its chemical composition is presented in Table 2. The abovementioned composites were manufactured via the squeeze casting method. The properties of the utilized Saffil^TM^ fibers (Alkegen CHQ—Buffalo, New York, NY, USA) and nickel-coated graphite flakes (Thermo Fisher Scientific, Warsaw, Poland) are displayed in Table 3 and Table 4, respectively.

Among the commercially available nickel-coated and copper-coated graphite flakes, the nickel-coated ones were selected. Nickel coating offers better protection against corrosion, which is crucial in ensuring the long-term stability and reliability of the composite in various environmental conditions [48]. Additionally, nickel coating can improve mechanical properties and the adhesion of the graphite flakes to the AlSi12 matrix. Copper has a lower hardness compared to nickel, which may lead to poorer mechanical stability of the composite when compared to nickel coating [49,50,51]. Although copper-coated graphite flakes can improve heat transfer due to higher thermal conductivity compared to nickel-coated [52], the specific requirements of this study, which emphasized corrosion resistance and mechanical properties, made nickel coating the more suitable choice.

For sample preparation, commercially available as-cast AlSi12, produced via gravity casting, was utilized. This material was then remanufactured using a different method—squeeze casting—with parameters that were analogous to those described below for the composites. The main difference was in the preform preparation. Therefore, in the performed tests described below, the AlSi12 alloy after the squeeze casting process was used. The production of composite materials involved two principal stages: the creation of a preform, followed by its infiltration with aluminum alloy using the squeeze casting technique.

In the case of Composite I, the preform was prepared from δ-alumina Saffil fibers that were mixed in an aqueous, silica binder solution. To ensure the homogeneity of the mixture, it was stirred in a glass container using a laboratory mixer designed for molding sands for approximately 10 min, at a high rotor speed of about 5 rotation per second, until a homogeneous milky suspension was formed. This process appears to have effectively broken up the fiber bundles, ensuring good dispersion and uniformity of the mixture. After the homogeneous mixture had been achieved, the process of drying and forming the mixture into the required shape was performed. In the next step, the preform was fired at 950 °C and the binder effectively created robust connections between fibers, leading to mechanically and thermally stable preform structures. In the case of Composite II, in the first step of preform preparation, Saffil fibers and nickel-coated graphite flakes were mixed in the appropriate proportion. Additionally, the firing process was conducted in a CO2 atmosphere to minimize carbon oxidation.

In the final infiltration process, the AlSi12 alloy was remelted to a temperature of approximately 720 °C. The preforms were preheated to 500 °C and were put into the mold shortly before pouring. In preparation, the primary components of the mold—the die and the punch—were mounted on a hydraulic press and preheated to temperatures of 300 °C and 200 °C, respectively. The melt was squeezed for 15 s at 150 MPa pressure [33,37].

**Figure 1:**
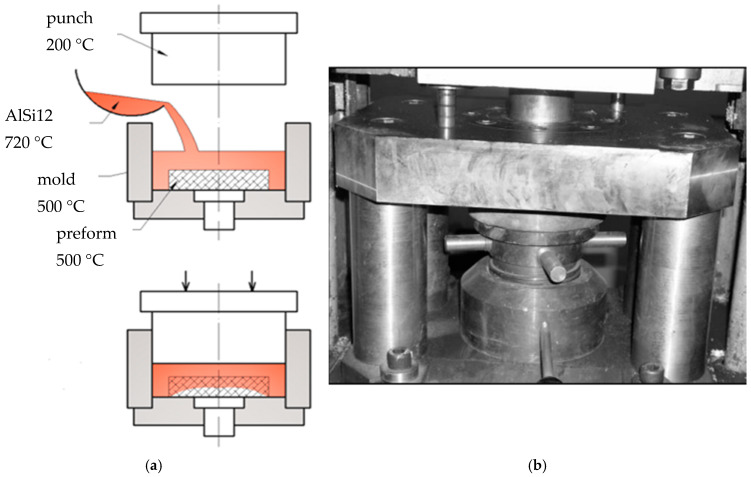
Squeeze casting process setup—the illustration (**a**) and the equipment (**b**) [53,54].

The experimental setup used for the squeeze casting process were provided in Figure 1 below.

### 2.2. Microstructural Analysis

The microstructure was characterized via SEM image analysis utilizing the Hita-chi TM3000 scanning electron microscope with the EDS/EDX system (Hitachi High-Technologies Corporation, Tokyo, Japan). This analysis was conducted in order to present the structure of the investigated materials and to identify particular phases. Furthermore, after flexural strength testing, additional images of fractures were produced and described.

Before analysis, the samples were polished using different grades of emery paper up to 4000 grit. Additionally, in the next step, the test pieces were felted. Afterwards, the materials were cleaned in distilled water and air-dried. It must be added that this sample preparation process did not apply to fractures.

### 2.3. Mechanical Properties

In order to investigate the effect of the composite reinforcement relative to the matrix base alloy, a flexural test was performed on three sample types: Composite I, Composite II and pure matrix alloy—AlSi12, four samples of each material.

Mechanical properties were evaluated using the Instron 5944 universal testing machine, model no. 5944L3909 (Instron, High Wycombe, UK), equipped with a 2 kN load cell. Test samples were rectangular beams with cross-section dimensions of 2 × 5 mm and a length of 40 mm. The test procedure was developed based on the flexural strength test procedure specified in ASTM C1161-18 [55]. Due to the shape and size of the manufactured composite, samples were made smaller but with maintained critical dimension ratios. Samples were preloaded with a 50 N load and then tested with a loading speed of 1 mm/min until a fracture occurred or a significant loading force drop due to plastic deformation. The support span was set as 16× sample thickness, which is 32 mm.

Moreover, Brinell hardness (HBW 2.5/31.25) testing was conducted on samples for better material characterization. For each sample, the measurement was taken from five different points and then the hardness value averaged. This investigation was performed using a Brinell hardness tester (VEB “Fritz Heckert”, Leipzig, Germany).

### 2.4. Electrochemical Measurements

To compare corrosion resistance of the tested materials in a 3.5% NaCl aqueous solution at room temperature, potentiodynamic polarization (LSV—linear sweep voltammetry) and electrochemical impedance spectroscopy (EIS) using a potentiostat (Metrohm Autolab BV, Utrecht, The Netherlands) working in a three-electrode system with Ag/AgCl (3M KCl) as a reference electrode were employed. The EIS spectra measured at OCP with the amplitude of the signal equaled 10 mV and a frequency range from 100 kHz to 50 mHz [56]. To propose electrical equivalent circuits and to determine their parameters, the NOVA 2.1 program was employed.

Before the measurements, the samples were polished using different grades of emery paper up to 4000 grit. Afterwards, the test pieces were cleaned in distilled water and air-dried. Finally, the materials were immersed in ethanol for 5 min in an ultrasonic cleaner and again air-dried. Due to the limited amount of produced composites and the need to prepare multiple specimens for destructive mechanical testing, only one sample was examined for each material. It should be added that the goal of conducted electrochemical measurements was only to evaluate the influence of used reinforcements on change in corrosion properties.

## 3. Results

### 3.1. Microstructural Analysis

**Figure 2:**
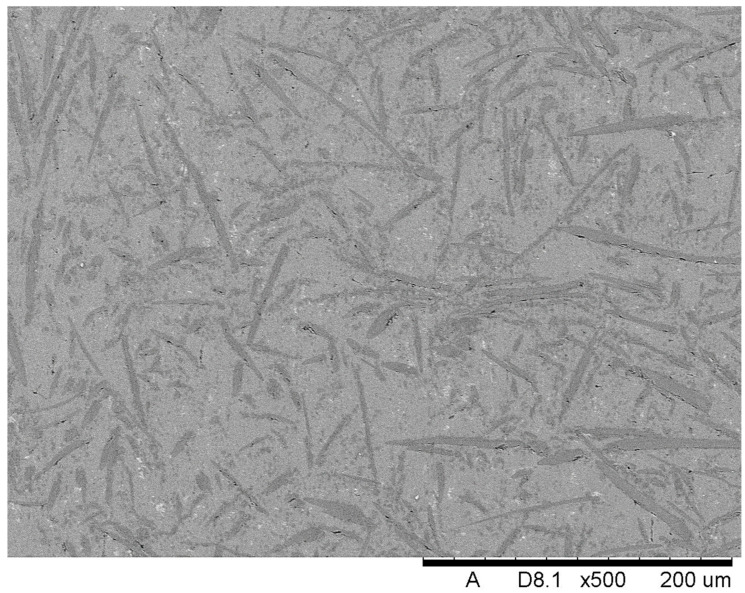
The microstructure of Composite I reinforced with ceramic Saffil fibers.

The SEM investigation was conducted in order to present and evaluate the structure of the obtained composites. Figure 2 below displays the structure of Composite I, where ceramic Saffil fibers arranged randomly in all directions are clearly visible. Based on the conducted SEM analysis and referring to the performed production process and prepared preform, it should be stated that the reinforcement is distributed uniformly in the matrix.

Homogenization of the material is indeed desirable, because it ensures the uniform distribution of properties, including mechanical ones, throughout the entire volume of the composite. A uniform distribution of reinforcing fibers in the matrix is expected to enhance the mechanical performance and overall consistency of the material. This finding is consistent with other studies in the literature, which have shown that uniform fiber distribution in composites improves both the material’s strength and durability [21,22,23].

**Figure 3:**
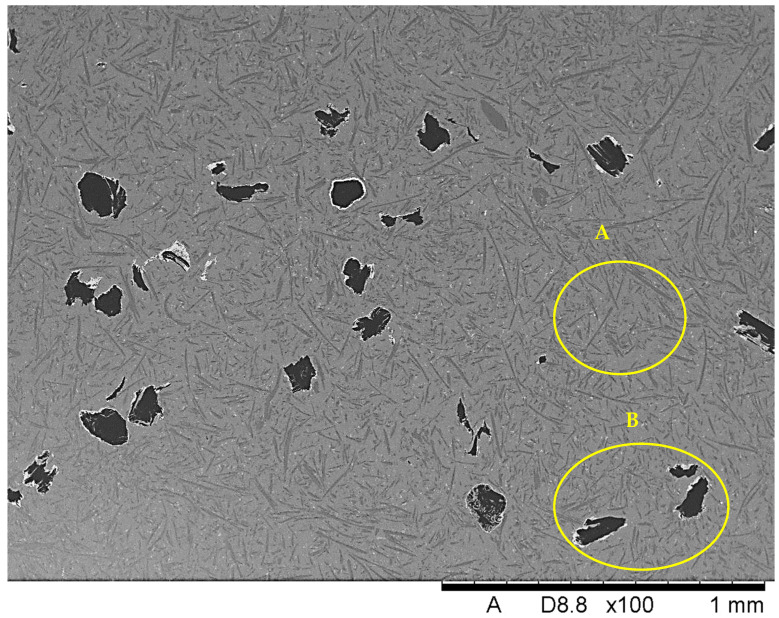
The microstructure of Composite II reinforced with ceramic Saffil fibers (A) and nickel-coated graphite flakes (B).

**Figure 4:**
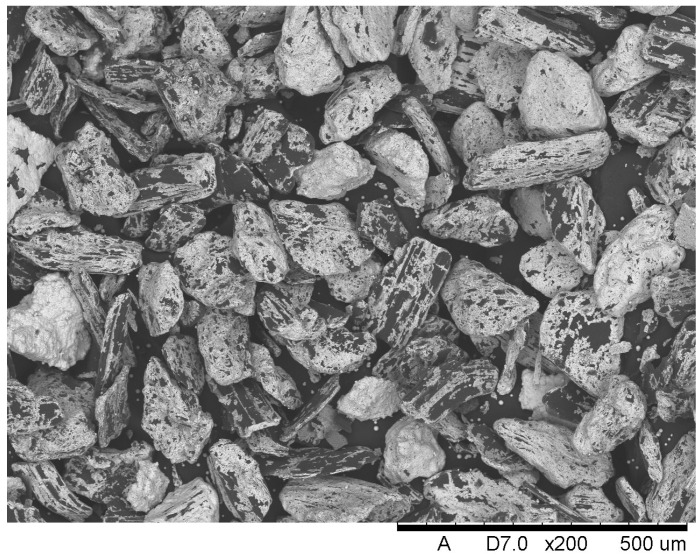
Nickel-coated graphite flakes used in the manufacturing process of Composite II.

Figure 3 provides the microstructure of Composite II strengthened with Saffil fibers and nickel-coated graphite flakes, which are displayed in Figure 4. SEM images showed poor adhesion between nickel and graphite, indicating a distinctly weak bond between the phases. The nickel-coated particles tend to detach easily from the graphite flakes. In Figure 4, nickel powder can be seen deposited on the black adhesive tape used to mount the sample to the steel holder. Furthermore, it should be noted that the nickel layer does not form continuous and uniform coverage. Portions of the graphite surface remain exposed, as evidenced by the clearly visible dark carbon regions. This partial coating may influence the properties of the final composite material. Based on the SEM images, it can be stated that the shape of the graphite flakes can be described as irregular with plate-like characteristics. The particle size was estimated to be 150 ± 45 μm, which represents the average of 20 measurements. The maximum dimension of each flake was used for the calculations. The minimum dimension in the analyzed statistical sample was about 50 μm, while the maximum dimension was about 280 μm.

**Figure 5:**
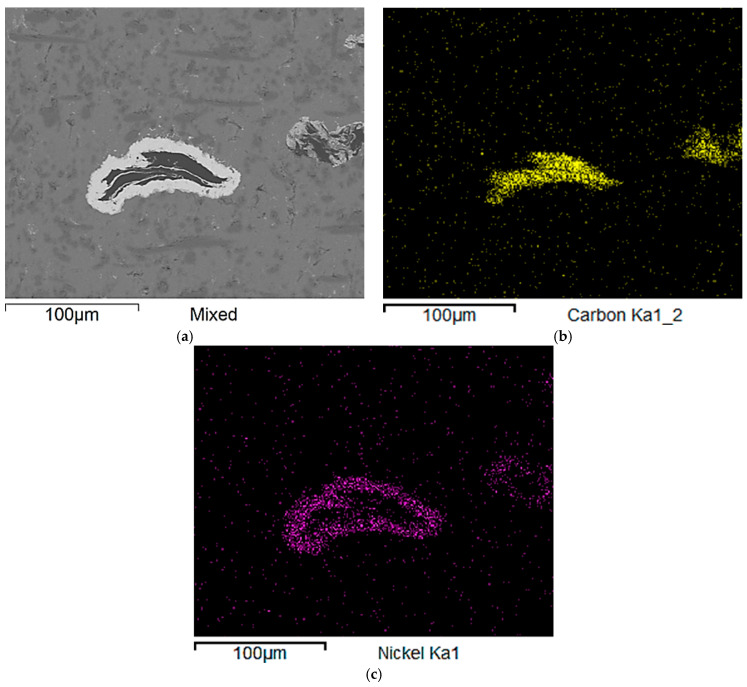
The microstructure of Composite II—selected area with a graphite flake (**a**) and element maps providing carbon (**b**) and nickel (**c**).

The selected region from the microstructure of Composite II (Figure 3) was investigated in terms of element mappings in order to detect particular elements creating the employed flakes (Figure 5). As expected, the results of this analysis provide carbon (main component) and the surrounding nickel (component of the protective coating).

### 3.2. Mechanical Properties

**Figure 6:**
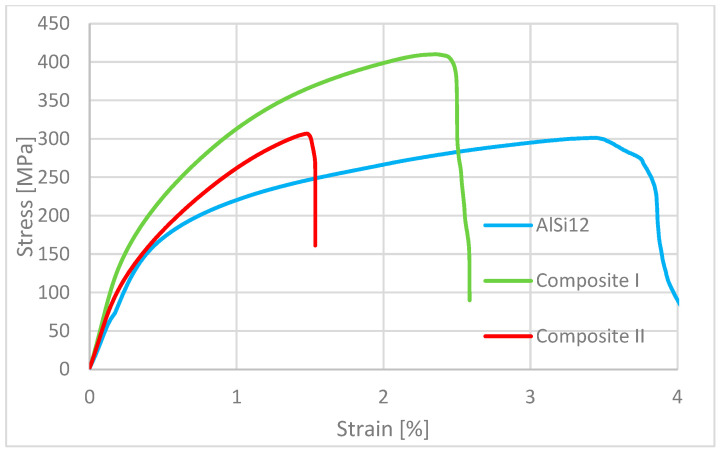
Representative stress–strain characteristics of flexural strength for raw matrix—AlSi12, Composite I and Composite II.

**Figure 7:**
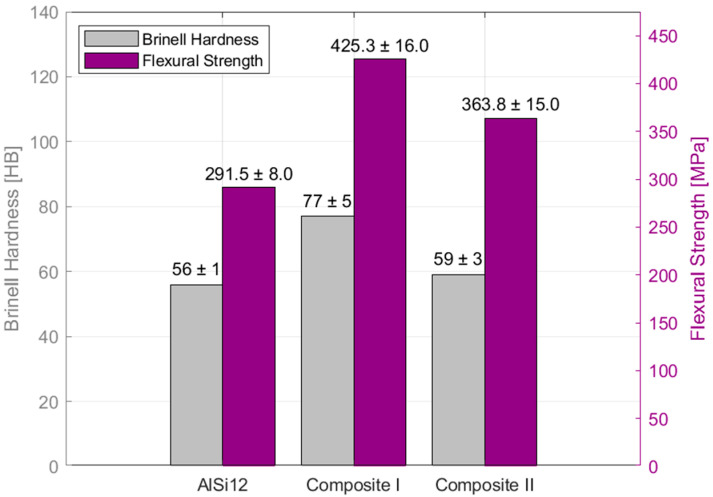
Collected flexural strength and Brinell hardness for matrix alloy AlSi12, Composite I and Composite II—averaged values.

Mechanical investigations revealed that both the base matrix alloy and the composites exhibit similar stress–strain characteristics, with a clear elastic region transitioning into plastic deformation. As shown in Figure 6, the pure matrix material (AlSi12) demonstrates a distinct transition, while Composites I and II show a more gradual transition. This is likely due to the uniform load distribution provided by the alumina reinforcement throughout the matrix. Furthermore, a reduced deflection was observed in the composite materials at the point of maximum stress, a behavior typical of such composites due to the addition of hard ceramic particles which decrease ductility [48]. This increased stiffness is reflected in the flexural modulus of elasticity, which was measured at 37.6, 70.2, and 64.5 GPa for AlSi12, Composite I, and Composite II, respectively. As depicted in Figure 7, Composite I exhibited the highest average resistance to bending loads (425.3 MPa), while AlSi12 showed the lowest (291.5 MPa).

Increase in mechanical parameters for Composite I can be induced by synergy of several strengthening mechanisms operating simultaneously. The high hardness is predominantly the result of Thermal Mismatch Dislocation Hardening (Δσ_CTE_), where the CTE (Coefficient of Thermal Expansion) difference between the AlS12 matrix and the alumina reinforcement generates an exceptionally dense network of strengthening dislocations according to Taylor’s theory by inducing complex stress state in the material where ceramic fibers are in compression and metal matrix is in tension. The high flexural strength is achieved through a combination of effective Load Transfer (Δσ_LT_) to the continuous, high-modulus fibers (fibers R_m_ can reach 6–11× higher value than matrix) and the crucial Microstructural Modification of the AlSi12 matrix, which refines the eutectic silicon phase, thereby increasing the intrinsic fracture resistance of the material [57,58,59,60].

These values are slightly lower than those reported by J. Maj et al. [47]; however, variations in composite manufacturing processes can account for such differences. Composite II demonstrated a significantly lower resistance to bending compared to Composite I, yet it was slightly higher than the base alloy. This is predictable, as the presence of graphite flakes tends to reduce the composite’s tensile and flexural strength, as well as its hardness. This weakening effect may be caused by poor interfacial bonding with the graphite flakes, which can introduce defects. Nevertheless, this trade-off is often accepted for the benefit of enhanced tribological properties.

Also in Figure 7, the presented averaged Brinell hardness seems to confirm the observations where the hardest material is Composite I due to the high content of hard alumina fibers; for Composite II, hardness is compromised due to the presence of graphite flakes; and the lowest hardness is seen for the pure matrix as there is no additional strengthening mechanism.

**Figure 8:**
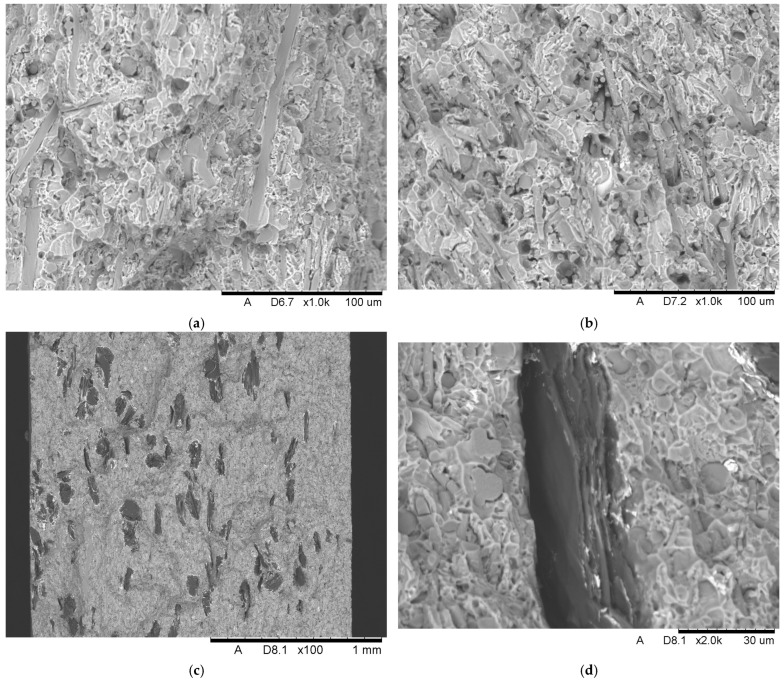
SEM images of fracture after flexural test of—Composite I (**a**,**b**) and Composite II (**c**,**d**).

After conducting the flexural strength test, fracture SEM images were produced (Figure 8). SEM analysis showed that Saffil fibers were fractured and broken after the failure of both composites, which clearly indicates a strong interfacial adhesion between the reinforcing fibers and the surrounding matrix material. Weak bonding would be indicated by a significant number of fibers pulled from the matrix. It should be noted that the fragile δ-alumina ceramic fibers fractured mainly orthogonally to their axes, with no pull-out effect, resulting in relatively flat fractures [33]. In the case of Composite II, Figure 8c provides an overall view of the fracture and shows a uniform distribution of graphite flakes, which implies satisfactory effectiveness of the stirring employed during the fabrication process. In the case of Composite I, the SEM images revealed a few small voids between the fibers that were not fully filled with the alloy. However, these regions appear only sporadically, thus it can be stated that the infiltration achieved acceptable penetration.

The research on AlSi12-based composites provides important insights into their mechanical properties. These materials could be useful in the automotive and aerospace industries, where strong mechanical performance is essential. By optimizing these properties through the right choice of reinforcement, materials with specific characteristics can be created to meet particular engineering needs. The ability to further adjust the mechanical properties of these composites can help improve the performance and durability of components used in demanding applications, such as structural parts in vehicles, aircraft or engines and turbines components, where both strength and resistance to harsh conditions are crucial.

### 3.3. Electrochemical Measurements

In order to compare the corrosion properties of the tested materials and to deter-mine the influence of the utilized reinforcements on the corrosion resistance of the manufactured composites, LSV and EIS measurements were performed. The obtained results were compared to values determined for the matrix alloy. The tests were con-ducted in a 3.5% NaCl aqueous solution at room temperature.

**Figure 9:**
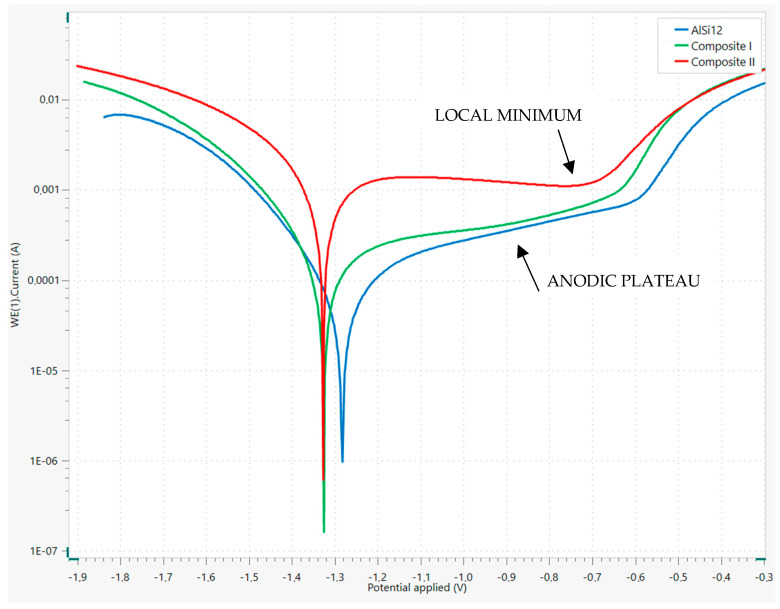
Potentiodynamic polarization curves of tested materials.

Figure 9 and Table 5 present the potentiodynamic polarization curves obtained during LSV measurements and the calculated corrosion parameters based on this chart using the Tafel analysis method, respectively. It should be mentioned that in the anodic regions there are areas corresponding to anodic plateau that implied difficulties with linear extrapolation of the Tafel regions. It was for these reasons that the linear polarization resistance method was performed in addition and the results were shown in Table 6. The corrosion rates calculated in these two techniques are very close to each other for each material. Furthermore, this did not affect the overall interpretation of the results or the final conclusions.

Based on the results provided in Table 5 and Table 6, it should be stated that the highest corrosion potential and the lowest corrosion rate were determined for AlSi12, which implies that the matrix alloy without any reinforcement demonstrates the highest corrosion resistance compared to the other materials under analyzed conditions. This means that the structure modifications—the introduction of Saffil fibers and graphite flakes—worsened the corrosion properties of the AlSi12 matrix. The same conclusion results from interpretation and comparison of polarization resistance value $R_{P}$, which indicates resistance of the material to the corrosion current.

It should be emphasized that the highest corrosion rate was observed for Composite II despite the application of the nickel coating on the used graphite flakes, intended to prevent the intensification of corrosion processes. This key parameter estimated both by Tafel and linear polarization resistance methods is one order of magnitude higher compared to the AlSi12 alloy and Composite I. Whereas, in these two cases, the difference is not significant. Therefore, it can be concluded that the addition of graphite flakes had a negative impact on corrosion resistance and the nickel coating did not fulfill its function.

It is worth mentioning that the corrosion potential values for both fabricated composites are very similar to each other. However, no far-reaching conclusions should be drawn on this basis. It should be noted that this parameter indicates the tendency of metallic elements to form oxidized compounds, which is associated with corrosion processes and results from the interplay of multiple factors—for instance, chemical composition of the matrix, percentage of reinforcement, properties of the oxide layer, properties of the hydroxide film, and extent of the specific surface area in contact with the electrolyte. The crucial parameter determining the corrosion rate is the current density, and the observed values of this parameter are dissimilar for Composite I and Composite II.

In Figure 9, in the cases of AlSi12 and Composite I, regions referring to anodic plateau, which indicates the ability of the materials to undergo passivation, can be observed. The curve obtained for Composite II in this area appears different. An initial increase in current is observed, culminating in a local maximum. This is followed by a decline to a local minimum, after which the current begins to rise again. This type of curve is typical for many divalent and trivalent metals (for instance iron, aluminum) and may result from formation of a passive film composed of sparingly soluble chemical compounds—for instance aluminum hydroxide. At this point, it may be assumed that in the cases of AlSi12 and Composite I the passive layer consists of a different compound, namely aluminum oxide. Therefore, it can be assumed that the differences in the properties of the formed protective layer influenced the values presented in the above tables and contributed to the low corrosion resistance of Composite II.

**Figure 10:**
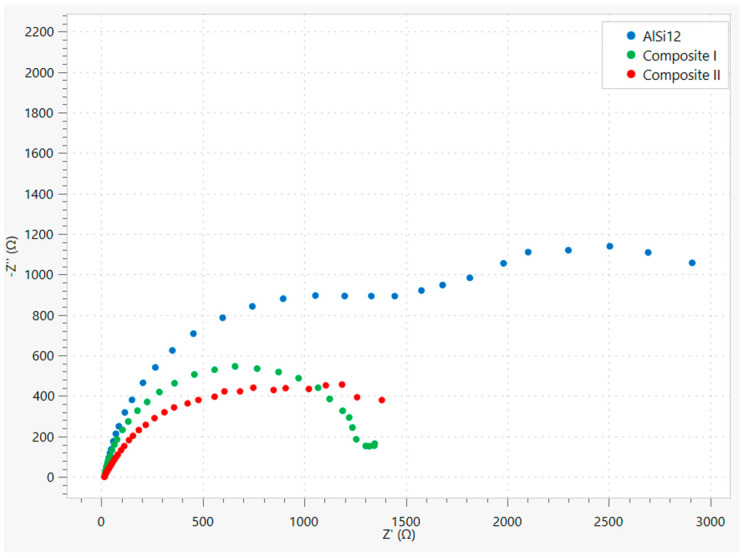
Nyquist plot for investigated materials prepared based on measurement data.

The EIS spectra were presented in the form of a Nyquist plot (Figure 10). In the next step, electrical equivalent circuits (EEC) were proposed for each corrosion system. When selecting the most suitable model, the diagrams employed in other articles were considered. In the work [61], Duygun et al. investigated unmodified and Sr-modified AlSi12 alloy in order to evaluate the influence of immersion time (1 h, 24 h, 72 h and 120 h) on corrosion behavior in a 3.5% NaCl solution. The authors decided to select two models under varying material and time-related variables—to be precise type [R(Q[R(QR)])] and type [R(Q[R(Q[RW])])]. Other models presented in works [62,63,64,65,66] were considered, including type [R(QR)(QR)]. In these articles, the authors also investigated the corrosion resistance of aluminum-silicon alloys and their modifications in an NaCl solution. All of the tested EEC models included two time constants. Some of them were more complicated and contained a Warburg impedance. Finally, the most suitable models for obtained measurement data were presented.

**Figure 11:**
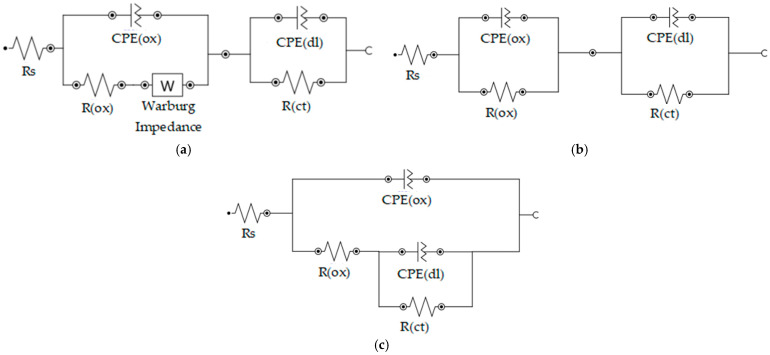
Equivalent electrical circuit (EEC) models proposed for investigated material—AlSi12 type [R(Q[RW])(QR)] (**a**), Composite I type [R(QR)(QR)] (**b**) and Composite II type [R(Q[R(QR)])] (**c**).

Figure 11 presents the proposed equivalent electrical circuits for the tested materials. The obtained measurements results required the proposal of distinct models for each case. The resistance of used electrolyte is represented by the symbol Rs. The systems with two time constants had to be considered in each case similarly to those in the investigations performed in the works cited above. However, simpler circuits were also tested. The first time constant includes capacity in the form of a constant phase element CPE(ox) and electrical resistance R(ox) of the protective oxide layer, which formed on the surface of the material. The second time constant reflects the double layer and consists of CPE(dl) and R(ct), analogously. Considering the diagrams in Figure 11a,b compared to Figure 11c it should be underlined that those elements are connected in different ways, which indicates differences in the properties of the oxide layer and the double layer, resulting in variations in electrical properties and corrosion resistance. Furthermore, the EEC proposed for AlSi12 includes the Warburg impedance, which indicates that the ongoing corrosion processes are also controlled by diffusion-related mass transfer through the passive layer. Nevertheless, it should be added that charge transfer control is present in each of the analyzed circuits.

It should be emphasized that for each investigated material and the measurements data obtained for it, the EEC model was selected and proposed with the lowest possible value of the coefficient $\chi^{2}$ (which evaluates the accuracy of the fit) and with the minimum values of the estimated errors for the elements of the circuit.

The estimated values of particular parameters for each material obtained during the simulation were provided in Table 7. Comparison may allow for the evaluation of corrosion behavior in the tested environment.

As mentioned above, the different connection between electrical elements in the proposed models is associated with the properties of the oxide and double layers. Furthermore, the results in Table 7 indicate that the highest value of R(ox) was estimated for the AlSi12 alloy and is three orders greater than for Composite I and six orders greater than for Composite II. A high R(ox) value is the indication of an oxide layer more resistant against the corrosion process associated with charge transfer and the attack of $Cl^{-}$ ions [61]. It should be added that this parameter was estimated with a relatively low error rate. It is also important to consider the N parameter that characterizes the constant phase element of the oxide layer. For N = 1, the CPE behaves as a pure capacitor, for N = 0.5 as an infinite Warburg impedance, and for N = 0 as a pure resistor [56]. The provided results show that for AlSi12 the value of this parameter is very close to u nit, which implies better protection against corrosion processes associated with electric charge transfer compared to manufactured composites. Whereas, the Y0 parameter describing the CPE and corresponding to the electrical capacity unit is difficult to compare due to the calculated value for Composite I, which was estimated with a relatively high error. This may be the result of the fact that for this material the N parameter is very close to zero, hence the CPE in this case can behave as a pure resistor. Nevertheless, it does not influence the general interpretation of the results because the key parameter in this case is the electrical resistance of the oxide layer represented by R(ox).

The comparison of the constant phase element of the double layer CPE(dl) is also difficult for a similar reason. In this case, the AlSi12 values were estimated with relatively high errors. However, it should be underlined that the EEC model with the lowest values of errors estimated for circuit elements was finally selected and presented in this work. But, based on the provided data, it can be noted that the values of particular parameters for Composite I and Composite II are very close to each other. Hence, it may be concluded that the double layers of these materials have similar properties despite the addition of graphite in the case of Composite II. This may mean that the Saffil fibers have the main impact on the structure of the double layer.

In the case of AlSi12 the most suitable EEC model included the Warburg impedance which results from the diffusion of ions within the surface layer. The presence of Warburg impedance indicates that corrosion processes are controlled by mass transfer phenomena and it may be the result of the natural oxide layer and/or corrosion product layer. Slow diffusion is observed with low Y0 values The lack of Warburg impedance in the cases of Composite I and Composites II indicates that the charge-transfer reaction is the only mechanism in the corrosion processes. It should be emphasized that the degradation of the oxide layer may be responsible for this behavior. Furthermore, it also indicates that this layer cannot block the attack of $Cl^{-}\;$ anions. Inhomogeneity of the oxide layer causes $Cl^{-}\;$ anions to adsorb/absorb in specific areas. A defective surface layer is more susceptible to pitting corrosion [61,67].

Based on this provided description, it can be stated that the protective oxide layer in the case of AlSi12 demonstrates the highest corrosion resistance among the tested materials. The worst protective properties were observed for Composite II. These conclusions confirm statements that were formulated based on LSV chart analysis (Figure 9).

**Figure 12:**
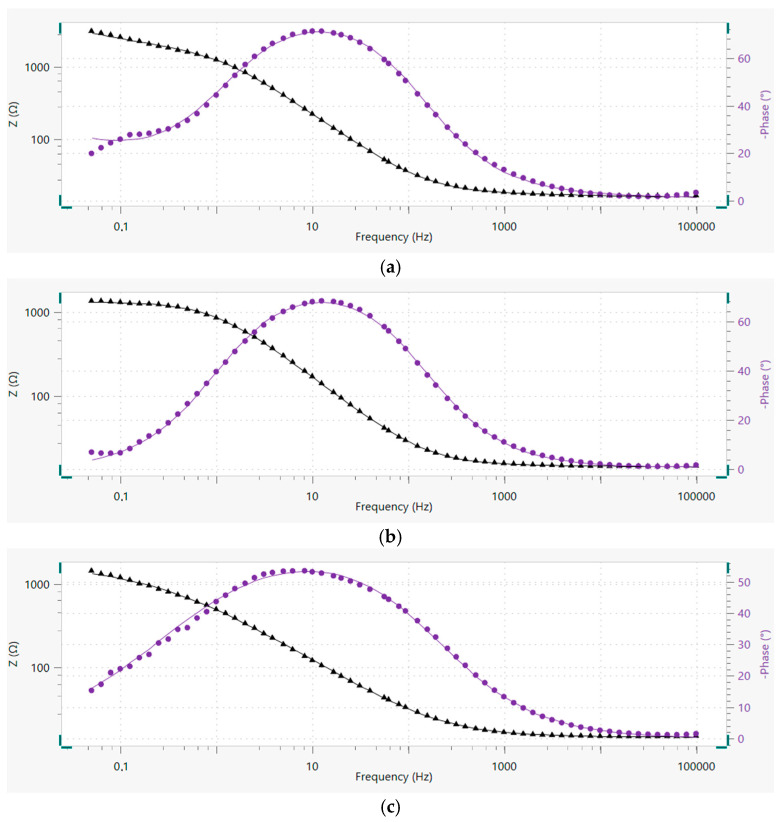
Bode plots with fitting lines for investigated material—AlSi12 (**a**), Composite I (**b**) and Composite II (**c**). The black color represents the magnitude, and the purple color represents the phase angle.

Additionally, in order to show the accuracy of the presented fit, Bode plots with fitting lines for each material are displayed in Figure 12.

The results of the EIS investigation confirm the findings obtained through interpretation of the LSV measurement data, including the Tafel analysis and the linear polarization resistance analysis. The electrochemical measurements indicate that the AlSi12 alloy demonstrates the highest corrosion resistance in a 3.5% NaCl solution at room temperature compared to other materials. The lowest corrosion resistance was observed for Composite II reinforced with Saffil fibers and nickel-coated graphite flakes. Therefore, modification of the matrix alloy through the addition of reinforcements caused weakness of the corrosion properties.

The explanation for this may be the properties of the protective oxide layer of the particular materials. The electrochemical tests indicate a higher effectiveness of the natural layer for the AlSi12 alloy and a limited effectiveness in mitigating the electrochemical corrosion processes in the case of Composite II.

It is very difficult to evaluate whether the nickel coating has successfully fulfilled its intended function because there is no comparison with any composite reinforced with pure graphite flakes under examined conditions. SEM investigation did not conform the presence of Al_4_C_3_. Nevertheless, the determined corrosion rates for Composite II are one order of magnitude higher compared to the other materials. Furthermore, SEM images showed poor adhesion between nickel and graphite, indicating a distinctly weak bond between the phases.

It should be emphasized that the samples were polished before the electrochemical measurements. Aluminum and its alloys naturally undergo passivation. However, for practical applications, the anodization process is often employed to artificially produce a surface layer with the required properties. At this point, further investigations could be proposed concerning the evaluation of the corrosion properties of manufactured composites after this additional process. Nevertheless, the primary objective of the presented work was to investigate the influence of the employed reinforcements on the corrosion resistance of the prepared materials.

## 4. Conclusions

In this work, composites based on the AlSi12 alloy reinforced with Saffil^TM^ fibers (Composite I) and with both Saffil^TM^ fibers and nickel-coated graphite flakes (Composite II) were developed using the squeeze casting method in the fabrication process. To evaluate their properties, investigations were conducted, including SEM analysis, flexural strength testing, Brinell hardness testing, LSV, and EIS. Electrochemical measurements were performed in a 3.5% NaCl solution at room temperature. Based on the above studies, the following conclusions can be formulated:

- The SEM investigations, conducted before and after the flexural strength test, confirmed the high efficiency of the squeeze casting process. The molten alloy successfully filled the spaces between the fibers during infiltration. The flakes were distributed homogeneously. Furthermore, the ceramic fibers fractured mainly orthogonally to their axes, without a pull-out effect, resulting in relatively flat fractures. This observation indicates a strong adhesion between the fibers and the matrix material. Unfortunately, SEM images revealed poor adhesion between nickel and graphite, highlighting a notably weak bond between the phases in this type of reinforcement. Nevertheless, the presence of Al_4_C_3_ was not observed.
- Manufactured composites exhibit a significant enhancement in flexural performance over the base matrix alloy for Composite I (flexural strength—425.3 MPa). This improved stiffness is attributed to the more effective and uniform load distribution provided by the alumina reinforcement. An increase in composite hardness confirms this effect.
- The inclusion of graphite flakes in Composite II resulted in a lower flexural strength compared to Composite I, which is consistent with the weakening effect of graphite particles. This reduction in strength is likely due to poor bonding between the graphite flakes and the Ni coating.
- Electrochemical measurements indicate that the corrosion resistance of the tested materials in the employed environment follows the order AlSi12 > Composite I > Composite II.
- The highest corrosion resistance was observed for the pure matrix alloy, which implies that the addition of reinforcements to the structure of the composites worsened the corrosion properties under the tested conditions. The highest corrosion rate was determined for Composite II, which was an order of magnitude higher compared to the other materials. The LSV and EIS results indicate that the explanation may be the differences in the properties of the protective oxide/hydroxide layer of the investigated materials. The LSV test showed that in the case of Composite II the external film may mainly consist of sparingly soluble and amorphous aluminum hydroxide. Furthermore, the EIS test and the proposed EECs indicated that the electrical resistance of the protective oxide layer follows the same order as the corrosion resistance. The difference between the AlSi12 and Composite I is three orders of magnitude, and the same difference is observed between Composite I and Composite II $\left(10^{3}>10^{0}>10^{-3}\right)$ in R(ox) parameter values.

## Figures and Tables

**Table 1:** Basic information about manufactured and investigated composites and proposed notations used in the article.

Proposed Designation	Matrix Alloy	Reinforcement
Composite I	AlSi12	20 vol.% Al_2_O_3_ Saffil Fibers
Composite II	AlSi12	20 vol.% Al_2_O_3_ Saffil Fibers + 3 vol.% nickel-coated graphite flakes

**Table 2:** Chemical composition of AlSi12 aluminum alloy employed in the investigation [47].

Element	Al	Si	Fe	Cu	Mn
**wt.%**	balance	10.5–13.5	0.55	0.05	0.35

**Table 3:** Properties of utilized Saffil fibers [32].

Composition [wt.%]	Density $\left[g/cm^{3}\right]$	Tensile Strength [MPa]	Young Modulus [GPa]	Diameter [µm]	Length [µm]
δ-Al_2_O_3_: 96–97 SiO_2_: 3–4	3.3	2000	300	2–4	0.1–0.3

**Table 4:** Selected properties of utilized graphite flakes coated by nickel [37].

Density $\left[g/cm^{3}\right]$	Young Modulus [GPa]
2.26	12

**Table 5:** Tafel analysis results describing corrosion behavior of tested materials in 3.5% NaCl solution.

Parameter	Unit	AlSi12	Composite I	Composite II
$E_{corr}$	[V]	−1.282	−1.325	−1.327
$j_{corr}$	$\left[\mu A/cm^{2}\right]$	16.3	31.6	149
$V_{corr}$	$\left[mm/year\right]$	0.56	1.01	4.77

**Table 6:** Linear polarization resistance analysis results describing corrosion behavior of tested materials in 3.5% NaCl solution.

Parameter	Unit	AlSi12	Composite I	Composite II
$E_{corr}$	[V]	−1.282	−1.325	−1.327
$j_{corr}$	$\left[\mu A/cm^{2}\right]$	11.7	24.4	142
$V_{corr}$	$\left[mm/year\right]$	0.39	0.78	4.57
$R_{P}$	[Ω]	642.44	308.07	52.89

**Table 7:** The values of the equivalent electrical circuits parameters estimated for the investigated materials using the fit and simulation tool.

Parameter	Unit	AlSi12	Composite I	Composite II
EEC model ^1^	–	(a)	(b)	(c)
Rs	[Ω]	14.4	11.7	14.3
CPE(ox)				
Y0 ^2^	$\left[\mu Mho\cdot s^{N}\right]$	97.0	$7.07\cdot 10^{3}$	235
N ^2^	[–]	0.924	0.192	0.411
R(ox)	[Ω]	$1.72\cdot 10^{3}$	3.85	$746\cdot 10^{-3}\;$
Warburg imp.				
Y0 ^3^	$\left[mMho\cdot s^{0.5}\right]$	1.11	–	–
CPE(dl)				
Y0 ^4^	$\left[\mu Mho\cdot s^{N}\right]$	$72.3\cdot 10^{3}$	151	326
N ^4^	[–]	0.145	0.884	0.746
R(ct)	[kΩ]	$1.10\cdot 10^{9}$	1.33	1.79
$\chi^{2}$	[–]	0.0490	0.0141	0.0298

^1^ Proposed electrical equivalent circuit (EEC) model for particular material (Figure 11); ^2^ Parameters describing CPE of oxide layer—CPE(ox); ^3^ Parameter describing Warburg impedance; ^4^ Parameters describing CPE of double layer—CPE(dl).

## Data Availability

The original contributions presented in this study are included in the article. Further inquiries can be directed to the corresponding author.
